# Exploring the Frontiers of Psychedelics: A New Chromatographic Method for Detection and Quantification of Psilocybin and Psilocin in *Psilocybe cubensis* Mushrooms

**DOI:** 10.1021/acsomega.5c02751

**Published:** 2025-07-10

**Authors:** Taynah Pereira Galdino, Lucas C. Oliveira, Mateus A. Luz, Raquel Costa Barbosa, Maria C. M. Torres, Katia Sivieri, Paula A. Fernandes, Márcio L. Santos, Antonio G. B. Lima, Suédina M. L. Silva, Marcus V. L. Fook

**Affiliations:** † Academic Unit of Materials Engineering, Federal University of Campina Grande, R. Aprígio Veloso, 882 − Bloco CJ3, Universitário, 58429-900 Campina Grande, PB, Brazil; ‡ Department of Chemistry, State University of Paraíba, R. Juvêncio Arruda, s/n, Bodocongó, 58429-500 Campina Grande, PB, Brazil; § Department of Food and Nutrition, Faculty of Pharmaceutical Sciences, R. Expedicionários do Brasil, 1621, Centro, 14801-360 Araraquara, SP, Brazil; ∥ Anhanguera University Center, R. Senador Fláquer, 456/459, Centro, 09010-160 Santo André, SP, Brazil; ⊥ Mechanical Engineering Department, 154624 Federal University of Campina Grande, R. Aprígio Veloso, 882, Universitário, 58429-900 Campina Grande, Brazil

## Abstract

Innovative therapies, such as psilocybin-assisted psychotherapies, hold great promises for treating anxiety, depression, and various other mental health disorders, addressing some of the challenges faced by conventional psychiatric medicine. This study focuses on developing and validating an HPLC-DAD methodology for detecting psilocybin and psilocin in extracts from psychedelic mushrooms intended for medicinal use. The methodology to has been validated following the guidelines of RDC No. 166/2017 from the Brazilian National Health Surveillance Agency (ANVISA). Utilizing a PerkinElmer C18 column (150.0 mm length × 4.6 mm internal diameter × 5 μm particle size), the gradient method employed ultrapure Milli-Q water (mobile phase A) and acetonitrile (mobile phase B), both acidified to 0.3% with formic acid (starting at a ratio of 95:5 v/v), with a flow rate of 0.8 mL/min over 18 min and an injection volume of 15 μL. The column temperature was maintained at 30 °C, and the analysis was conducted at a wavelength of 266 nm. The limit of detection (LOD) and limit od quantification (LOQ) values for psilocybin were 1.58 and 4.78 mg/L, respectively, while for psilocin, they were 1.70 and 5.17 mg/L. The method’s accuracy showed recovery intervals for psilocybin ranging from 80 to 120% and for psilocin from 98 to 116%. Accurately determining the content of psilocybin (2.57%) and psilocin (0.16%) is essential for their application as pharmaceutical compounds. This methodological rigor ensures that these substances can be reliably used in therapeutic contexts, underscoring the importance of precise quantification in developing safe and effective medical treatments.

## Graphical abstract

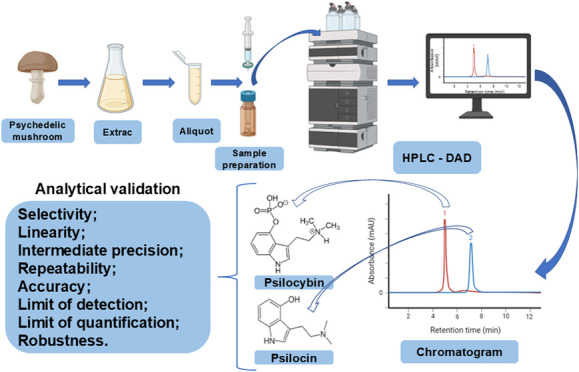

## Introduction

Psychedelics are a group of compounds found in living organisms (plants, mushrooms, cacti) as well as synthesized artificially (e.g., lysergic acid diethylamide - LSD). Despite their diverse molecular structures, these compounds share a significant characteristic: a hallucinogenic influence commonly associated with changes in mood, perception, and cognition.^1^ The use of psychedelics has a low potential for dependence, and unlike medications from the classes of opioids, sedative-hypnotics, and some stimulants, there is no evidence of neurotoxicity.^1,2^

Psilocybin (PSCB) and psilocin (PSC) are naturally occurring psychedelic compounds found in various species of mushrooms, notably *Psilocybe cubensis* and *Psilocybe semilanceata*. Chemically, psilocybin (4-phosphoryloxy-N,N-dimethyltryptamine) is a prodrug that is rapidly converted in the body to psilocin (4-hydroxy-N,N-dimethyltryptamine), the active molecule of this conversion exerts psychotropic effects (Figure 1). These compounds interact primarily with serotonin receptors in the brain, particularly the 5-HT2A receptor, leading to altered perception, mood, and cognition.^3^

**Figure 1:**
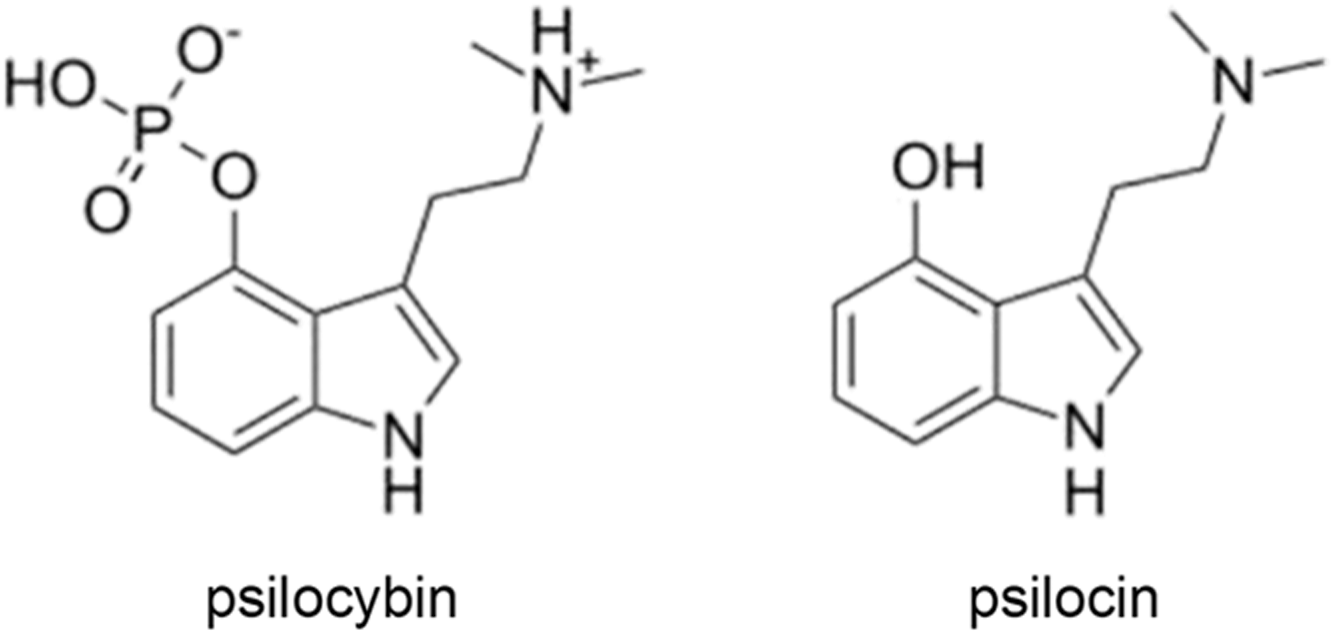
Chemical structure of psilocybin and psilocin.

Research indicates potential therapeutic benefits of psilocybin for mental health conditions such as depression, anxiety, and post-traumatic stress disorder (PTSD), likely due to its capacity to promote neural plasticity and modulate the brain’s default mode network. Clinical studies have shown psilocybin can induce profound and enduring changes in personality and emotional well-being, often described as mystical or spiritual experiences. Despite its characteristics, the use of psilocybin is regulated in many countries due to its potent psychoactive properties and the potential for misuse. Current research is focused on elucidating the mechanisms of its therapeutic effects and establishing protocols for safe and effective medical use.^4,5^ Additionally, psilocybin has low toxicity, showing no specific signs in isolated organs and is non-neurotoxic.^6–12^

However, from a legal standpoint, psilocybin and psilocin are included in the List of Prohibited Psychotropic Substances in Brazil, and there are no standardized methodologies for their identification and quantification. Detection and quantification of these substances are usually performed using high-performance liquid chromatography (HPLC). For analyzing the substances of interest using HPLC, various detection modes are employed, including diode array detectors (DAD), fluorescence detectors, electrochemical, voltammetric, and mass spectrometry detectors.^13–17^

To achieve pharmaceutical application objectives, it is necessary to establish and validate a chromatographic identification and quantification methodology for psilocybin and psilocin obtained from psychedelic mushrooms. In this study, we report the development and validation of an innovative HPLC-DAD method to identify and quantify psilocybin and psilocin in psychedelic mushroom extracts for medicinal use. Herein, the development and validation of the analytical method using HPLC-PDA were conducted following the norms and guidelines of the Resolution of the Collegiate Board of the National Health Surveillance Agency – ANVISA (Brazil), RDC No. 166/2017, which provides for the validation of analytical methods.^18,19^

The objective of this study was to develop a method to detect and quantify psilocybin and psilocin, extracted from *Psilocybe cubensis* mushrooms, with the purpose of enabling their dosage in pharmaceutical formulations for commercial and clinical use applied to psychedelic-assisted therapy. A simple and relatively low-cost extraction and analysis protocol was developed for reliable determinations of the substance contents. Method validation according to ANVISA RDC 166/2017 evaluates its detection and quantification limits, accuracy, precision and recovery to determine the reliability of the results in quantifying these analytes via HPLC-DAD.

## Materials and Methods

### Reagents and Standards

Psilocybin (1 mg/mL, Lipomed Document QC-CA-411L1) and psilocin (1 mg/mL, Lipomed Document QC-CA-410L1) standards were obtained from LAS do Brasil (Aparecida de Goiânia, Brazil). Batches of HPLC-grade acetonitrile (ACN) were sourced from Êxodo Científica (Sumaré, São Paulo, Brazil) and SK Chemicals (Seongnam, South Korea), and formic acid P.A. was obtained from Neon (Suzano, Brazil). Ultrapure water (18.2 MΩ·cm, TOC < 10 ppb) was produced using a Milli-Q system (MERCK, model eq 7000, Darmstadt, Germany). For sample preparation, 2 mL vials with a 9 mm diameter for HPLC (PerkinElmer, Connecticut), 0.45 μm polytetrafluoroethylene (PTFE) membrane filters from Unifil, and an analytical balance (Marte/Shimadzu, model AUY220, Kyoto, Japan) were used. The mobile phases (acetonitrile and ultrapure water, both acidified to 0.1% with formic acid P.A.) were filtered using a 1000 mL HPLC filtration system from Biocentrix, with hydrophilic cellulose nitrate membrane filters (0.22 μm pore size, 47 mm diameter) and hydrophilic nylon membrane filters (0.45 μm pore size, 47 mm diameter) from Merck Group, using a vacuum pump (Sppencer Scientific, Hipper Química model HQ 260, São Paulo, Brazil).

The *Psilocybe cubensis* mushrooms used in this study were imported into Brazil (Rosehill Apothecary Ltd., Rose Hill, Jamaica) through an import permit granted by ANVISA (AI 067/2025), specifically for this project, in accordance with the Special Authorization for the study of psilocybin and psilocin (AE No. 30/2024), which includes the control, storage, and handling of these substances.

The psychedelic mushrooms were collected from the wild, cleaned, and dehydrated in an air-circulation oven (Solab, model SL-102, São Paulo, Brazil) at 100 °C for 24 h, then ground using a mortar and pestle with the aid of liquid nitrogen (−196 °C). The extractive solvents used were ethanol P.A. from Química Moderna (Barueri, Brazil) and hexane P.A. from Neon (Suzano, São Paulo, Brazil), and the solubilization process was assisted by an ultrasonic bath (Ultronique, model Q9.5/40a̲, São Paulo, Brazil).

The entire research was conducted under a special authorization (AE) granted by the National Health Surveillance Agency (ANVISA, Brazil) for the storage, handling, and development of research involving prohibited substances (AE No. 30/2024).

### Extraction Protocol

Dried psychedelic mushrooms intended for extraction must reach the form of a fine powder. For this, the material must be placed in a mortar and submerged in liquid nitrogen, crushed with the help of the pestle until a powder mixture is formed. This material must be removed for sieving, passing through a sieve with a particle diameter of 170 mesh until all the material has a particle size smaller than 0.088 mm (170 mesh).

The extraction process consists of adding the mushroom powder to an extractive solution, composed of ethyl alcohol acidified to 2% acetic acid, in the proportion of 4 mL for every 100 mg of material. The mixture of material and solvent must be placed in amber-colored containers and sent to an ultrasonic bath (Ultronique, Q p.5/40A, São Paulo, Brazil) for 30 min. After the extraction process, the material is filtered through a vacuum filtration system, consisting of a pump coupled to a kitassato and a buckner funnel, until the solvent is completely removed. The solid material is used to reproduce the extraction process, performed in quadruplicate, following the same methodology, to ensure complete removal of the substances. The solvent obtained from the extraction processes is homogenized and evaporated in a rotary evaporator (Fisaton, Model 802, São Paulo, Brazil) under vacuum at a temperature of 40 °C, to concentrate the substance in the solvent (reaching volumes smaller than 100 mL), and then sent to an oven with air circulation (Solab, SL-10, São Paulo, Brazil) at 40 °C for complete removal of the solvent.

The dry extract is subjected to the removal of nonpolar compounds, through affinity extraction. For this, hexane is added to the dry extract in a ratio of 1:20 (g/mL), and subjected to ultrasonication for 30 min. After completion of ultrasonication, the hexane supernatant is discarded and the process is repeated in triplicate, with solvent renewal, taking the solid material to the oven with air circulation at 40 °C to remove the remaining solvent, obtaining the extract containing psilocybin and psilocin.

### Instrumentation and Experimental Procedure

The experiments were conducted using a high-performance liquid chromatography (HPLC) system from PerkinElmer (Shelton, Connecticut), model LC-FLEXAR, with a diode array detector (DAD), coupled with a SOGEVAC vacuum pump model SV40BI. This system was used for the development and validation of the analytical method to evaluate the fungal extract and the standards of psilocybin and psilocin.

An initial method, referred to as Method A, was developed as the first attempt to assess the presence of psilocybin and psilocin in the fungal extract of the hallucinogenic mushroom at concentrations of 25, 250, and 2500 mg/L. A PerkinElmer C18 column (250 mm length × 4.6 mm internal diameter × 5 μm particle size) was used. The gradient method employed ultrapure Milli-Q water as mobile phase A and methanol as mobile phase B (H_2_O starting at a ratio of 5:95 v/v, ending at 95:5 v/v), with a volumetric flow rate of 0.5 mL/min over 30.1 min and an injection volume of 10 μL. The column temperature in the oven was maintained at 30 °C, and wavelengths of 266 and 280 nm were analyzed.

Following that, a method named Method B was developed, stemming from the improvement of Method A, with samples at a concentration of 25 mg/L. A PerkinElmer C18 column (250 mm length × 4.6 mm internal diameter × 5 μm particle size) was utilized. The gradient method employed ultrapure Milli-Q water as mobile phase A and acetonitrile (ACN) as mobile phase B, both acidified to 0.1% with formic acid (H_2_O (0.1% formic acid): ACN (0.1% formic acid) starting at a ratio of 95:5 v/v), with a volumetric flow rate of 0.8 mL/min over 30.1 min and an injection volume of 10 μL. The column temperature in the oven was maintained at 30 °C, and wavelengths of 266 and 280 nm were analyzed.

Finally, Method C, resulting from the refinement of Methods A and B, was developed and utilized to identify, quantify, and validate the analytical method obtained, analyzing samples with a concentration of 25 mg/L. A PerkinElmer C18 column (150 mm length × 4.6 mm internal diameter × 5 μm particle size) was used. The gradient method employed ultrapure Milli-Q water (mobile phase A) and acetonitrile (mobile phase B), both acidified to 0.3% with formic acid (H_2_O (0.3% formic acid): ACN (0.3% formic acid) starting at a ratio of 95:5 v/v), with a volumetric flow rate of 0.8 mL/min over 18 min, and an injection volume of 15 μL. The column temperature in the oven was maintained at 30 °C, and the analysis was conducted at a wavelength of 266 nm.

All mobile phases were prepared by filtration through nylon membranes (pore size: 0.45 μm) and cellulose nitrate membranes (pore size: 0.22 μm) installed in a vacuum pump from Sppencer Scientific (Hipper Química, model SP704–25, São Paulo, Brazil) and degassed by sonication in an ultrasonic bath. Data acquisition and analysis were performed using Chromera Speciation software from PerkinElmer (Shelton, Connecticut).

### Sample Preparation

Initially, eight solutions containing psilocybin and psilocin standards at concentrations of 1, 5, 10, 25, 50, 75, 100, and 125 mg/L were prepared for linearity, selectivity, limit of detection (LOD), and limit of quantification (LOQ) studies. From these eight concentrations, five concentrations were chosen for the construction of the calibration curve, performed in triplicate, obtaining peak area responses for each substance.

For robustness, repeatability, intermediate precision, and accuracy assays, triplicate solutions containing psilocybin and psilocin standards at concentrations of 10, 50, and 100 mg/L were prepared. For intermediate precision, the analysis days and analysts who performed the analysis on each day were varied.

For the matrix effect study, eight solutions containing psilocybin and psilocin at concentrations of 1, 5, 10, 25, 50, 75, 100, and 125 mg/L were prepared. However, the solution with 1 mg/L concentration of Psilocybe cubensis mushroom extract was used as a matrix for the samples, in order to compare solutions without the extract matrix and with the extract matrix.

### Method Validation

#### Selectivity

The selectivity analysis was conducted in accordance with RDC No. 166/2017 guidelines for chromatographic method validations. These guidelines outline parameters that can be calculated from the chromatogram, in orden, to determine the selectivity of a method, such as resolution and chromatographic peak purity. The chromatographic equipment software automatically calculates these parameters, providing a clear indication of the method’s selectivity.

For resolution, the goal is to achieve a resolution of at least 1 between the peak of interest and the neighboring peaks. For chromatographic peak purity, the quality of the chromatogram is evaluated based on the type of detector used, with a peak purity value ranging from 1 to 2 indicating a pure peak.

#### Linearity, Limit of Detection (LOD) and Limit of Quantification (LOQ)

The linearity of the method is directly related to the ability to generate results linearly proportional to the concentration of the substance, being determined by the construction of a calibration curve with five points, analyzed in triplicate for psilocybin and psilocin, and evaluated by the linear regression method for the entire range established by the method. For this, eight different concentrations will be evaluated (1, 5, 10, 25, 50, 75, 100, and 125 mg/L) to construct the calibration curve, being evaluated through the linear regression of *y* = *f*(*x*), estimated by the least-squares method, including the linear association between the variables through the correlation coefficient (*r*), angular coefficient and the sum of the square of the residuals.

Based on the equations provided by the standard and data obtained from the calibration curve, it is possible to determine the lowest amount of analyte that can be detected (LOD) and quantified (LOQ), as indicated in eqs 1 and 2. This calculation used the slope and intercept data, together with the standard deviation and mean obtained from the calibration curve.

1 $$\mathrm{LOD}=\frac{3.3\times \sigma }{\mathrm{IC}}$$

2 $$\mathrm{LOQ}=\frac{10\times \sigma }{\mathrm{IC}}$$

where IC represents the slope of the calibration curve, and σ is the standard deviation.

#### Recovery, Accuracy and Precision

Recovery was evaluated at three concentration levels (10, 50, and 100 ng/mL) by spiking a standard solution of PSCB ans PSC into extract mushroom. For each concentration, three replicates were processed and analyzed. The analyte concentration obtained from the spiked matrix samples was compared to the theoretical value and corrected for any background signal detected in nonfortified matrix samples (blank extract).

Precision was evaluated by assessing the closeness of results obtained from assays with samples at known concentrations, as described in eq 3. To analyze intermediate precision, accuracy, and repeatability, the analysis was conducted on different days and by different analysts, while maintaining the same sample concentration levels and performing the analyses in triplicate.

3 $$\mathrm{SD}=\frac{\sigma }{\mathrm{CMD}}\times 100$$

where SD stands for relative standard deviation, σ is the absolute standard deviation, and CMD represents the average determined concentration.

#### Matriz Effect

To ensure the absence of matrix effects, it is essential to evaluate the concentration–response lines of the calibration curves for standards and fortified samples, assessing their parallelism. Parallelism indicates the absence of interference from matrix constituents, with a significance level of 5% applied in hypothesis testing. Therefore, the matrix effect study involved constructing curves for psilocybin and psilocin using samples at concentrations specified in the calibration curve, incorporating a matrix consisting of a 1 mg/mL solution of the extract. By comparing solutions with and without the extract matrix, the impact of this matrix in the proposed analytical method can be determined.

#### Working Range

The working range is established based on the data and results of linearity, precision, and accuracy. It is generally limited at the lower end by the limits of detection or quantification and at the upper end by the constraints imposed by the equipment. The working range (Ft) is calculated using the total concentration of the solution (*C*_t_), extract concentration (*C*_ext_), and theoretical concentration (*C*_theo_), as shown in eq 4.

4 $$\mathrm{WR}=\frac{C_{t}-C_{\mathrm{ext}}}{C_{\mathrm{theo}}}\times 100\%$$

The working range was calculated using the calibration curve equation obtained after application of the linear regression method. The extract concentration was determined from injections made to assess the matrix effect. Blank samples (containing only the extract) were injected, and data from the areas of the peaks of interest were collected. The areas were then calculated based on the equation derived from the matrix effect curves.

#### Robustness

The robustness of the developed method was assessed by analyzing its ability to tolerate small, deliberate variations in analytical conditions. The method was evaluated by intentionally altering parameters such as temperature (30 and 40 °C), the pH of the mobile phase with increased acidification (0.2 and 0.3% formic acid), and the supplier/batch of acetonitrile solvent used in the mobile phase (Êxodo Científica, São Paulo, Brazil, and SK Chemicals, Seongnam, South Korea). These variations represent typical environmental conditions encountered during method application, serving the same purpose of evaluation.

## Results and Discussion

### HPLC-PDA Method Development and Optimization

The method, referred to as Method A, uses a mixture of ultrapure water and methanol (95:5, v/v) without acidification for pH modulation. Based on the experiments, it was unable to achieve significant separation of the compounds in the hallucinogenic mushroom extract. Given the acidic nature of the main alkaloid, psilocybin, due to its phosphate group, it likely remained in its deprotonated form in solution, impairing the overall quality of the chromatogram (Figure 2a). Meanwhile, psilocin, the other alkaloid of interest, typically present in low concentrations, did not produce an appreciable peak in the chromatogram of the crude extract. Furthermore, this version of the method failed to yield significant results for the tested concentration variations.

**Figure 2:**
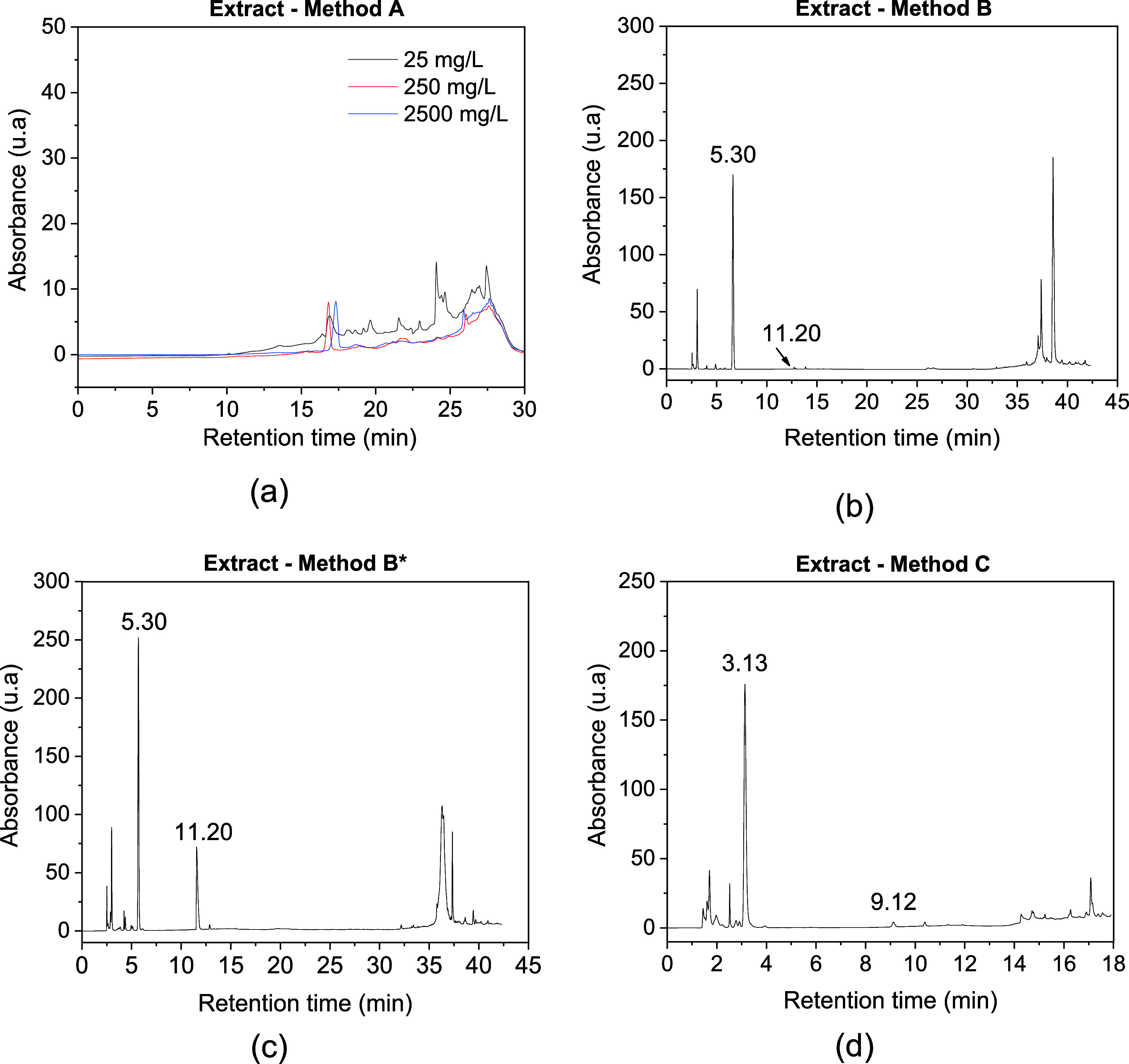
HPLC-UV chromatograms of (a) blank methanol using water:methanol (95:5 (v/v)), (b) water:acetonitrile both acidified to 0.1% with formic acid (95:5 (v/v)), (c) water:acetonitrile (acidified to 0.3% with formic acid) (95:5 (v/v)) and (d) water:acetonitrile (0.3% with formic acid) (95:5 (v/v)) (the time axis scale varies).

After evaluating the results obtained by Method A, necessary modifications were made to obtain analyzable chromatographic peaks, resulting in Method B. This method involves a longer run to account for the potential interference of other substances in the extract, ensuring accurate analysis of the desired compounds. The mobile phase was modified to a composition of ultrapure water and acetonitrile both acidified to 0.1% with formic acid. This change in the mobile phase generated a chromatogram with three well-defined peaks, suggesting the identification of psilocybin at 5.30 min and psilocin at 11.20 min; however, it was still not possible to precisely identify the substances due to variation in retention time across replicates (Figure 2b).

The mobile phases used were acidified to 0.3% with formic acid, to improve pH modulation, known as Method B*, and the analytical standards for both substances were analyzed using this method, allowing the determination of the retention times of the substances (Figure 2c). The identification was confirmed by the increase in the peak at 5.30 min for psilocybin and 11.20 min for psilocin, and the variability between analyses was minimized.

Finally, Method C involved replacing the 250 mm chromatographic column with a 150 mm column, as the extract contains few peaks of interest, none close to the analyzed substances. This change reduced the total chromatographic run time, improved analysis efficiency, and lowered solvent consumption without compromising result quality. The developed method for identifying psilocybin and psilocin has a total runtime of 18 min, with retention times of approximately 3.13 min for psilocybin and 9.12 min for psilocin, using ultrapure water and acetonitrile (95:5, v/v) both acidified to 0.3% with formic acid (Figure 2d). These retention times were confirmed by analyzing analytical standards of both substances.

### Validation of the Chromatographic Method

Based on Method C developed during the optimization phase, the validation process was initiated in accordance with the guidelines set forth by RDC No. 166/2017 of the Brazilian National Health Surveillance Agency (ANVISA).^18,19^

All assays prescribed by the standard were conducted, and all results were statistically evaluated using the specified parameters to ensure the quality of the obtained data. Stock solutions of the formulated standards were employed for the analysis under validation conditions. Consequently, a detection wavelength of 266 nm was selected and applied throughout the validation process, ensuring that all area values used in the calculations were derived from chromatograms recorded at this specific wavelength.

### Selectivity

The selectivity of the chromatographic method can be determined by assessing the purity of the peak obtained from injecting the pure substance, a characteristic closely linked to the peak’s resolution and symmetry factors. When evaluating this aspect for the substances using the method, it was observed that at all measured concentration levels, the peak purity consistently remains below 1.5. According to the specifications of the software used, this indicates high purity for the evaluated peak.

Furthermore, selectivity was assessed based on chromatographic resolution, a parameter calculated by the analytical software, with values above 1 considered acceptable. In all analyses of the extract containing psilocybin and psilocin, resolution values consistently exceeded the minimum specification, confirming the selectivity of the developed method. The chromatographic separation (Figure 3a) demonstrated well-resolved peaks for psilocybin and psilocin, indicating robust analytical specificity under the employed conditions. The UV spectral congruence between the mushroom extract and analytical standards (Figure 3b) further corroborates the identity of these compounds, as no significant deviations in absorbance maxima or spectral profiles were observed. This alignment, combined with peak purity assessments, confirms the absence of coeluting interferents, underscoring the reliability of the method for quantifying these psychoactive alkaloids in complex matrices. These findings validate the analytical approach and provide a foundation for future studies on psilocybin-containing fungi, emphasizing the importance of spectral verification in ensuring accurate compound identification.

**Figure 3:**
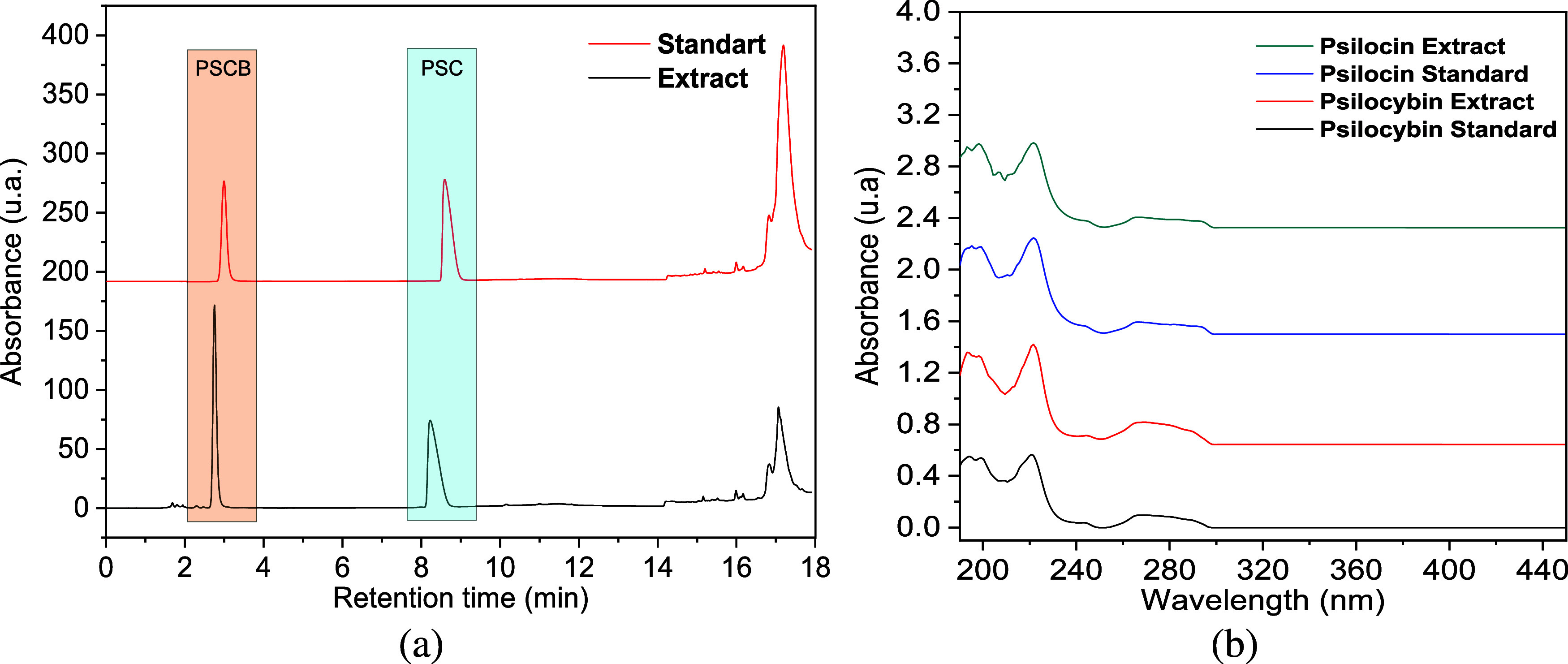
(a) Chromatogram of mushroom extract showing distinct peaks for psilocybin (PSCB) and psilocin (PSC) at retention times of 0–18 min, with absorbance monitored at 266 nm. (b) UV spectra (200–440 nm) of psilocybin and psilocin in the mushroom extract compared to their respective analytical standards.

### Linearity, Limits of Detection (LOD) and Quantification (LOQ)

The chromatographic method developed in this study produced calibration curves covering a working range from 5 to 125 mg/L for psilocybin (PSCB) and from 5 to 100 mg/L for psilocin (PSC) (Figure 4). Linear regression analyses were conducted by plotting the area against the concentration points used, as illustrated in Figure 5.

**Figure 4:**
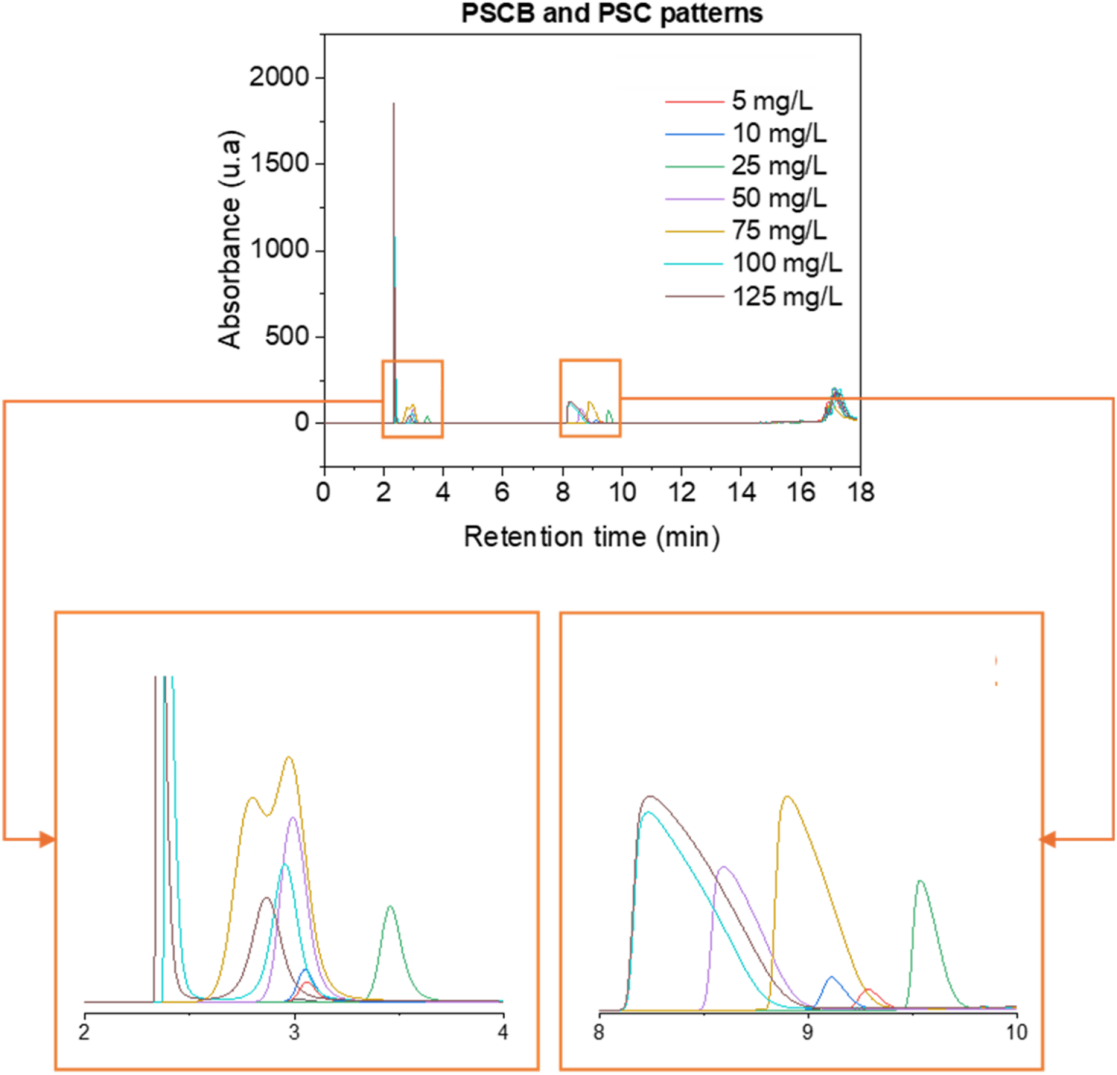
Chromatograms of injections for the development of the calibration curve of psilocybin and psilocin using different concentrations.

**Figure 5:**
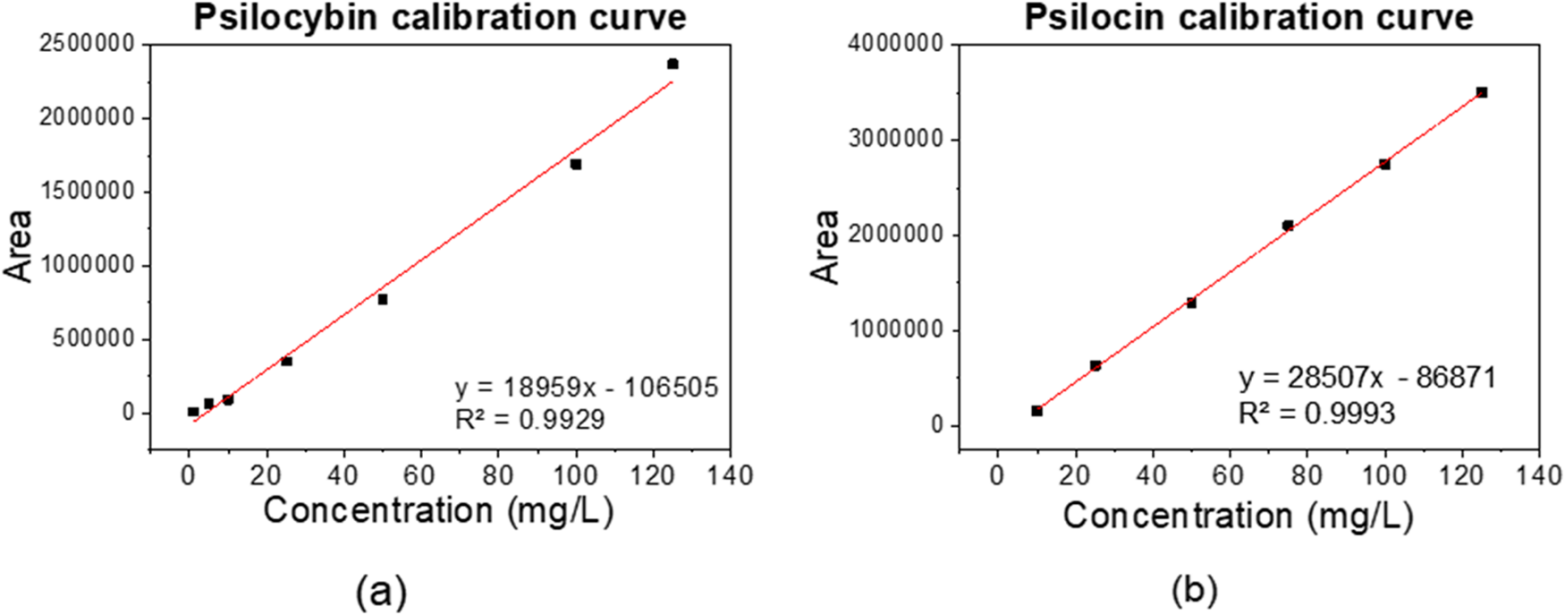
Calibration curves of working standards of psilocybin and psilocin in the concentration range of (a) 0–125 mg/L and (b) 10–125 mg/L.

The calibration curves were constructed using six concentration points: 5, 10, 25, 50, 100, and 125 mg/L for psilocybin, and 5, 10, 25, 50, 75, and 100 mg/L for psilocin (Figure 3). The linearity of the method was assessed through linear regression, evaluating parameters such as the y-intercept, slope of the regression line, coefficient of correlation (*R* ^2^), and sum of the squares of residuals. These results are presented in Figures 4 and 5. In Figure 5, the parameter *y* represents area and *x* represents concentration in mg/L.

According to the guidelines outlined in RDC No. 166/2017 from Anvisa (Brazil), both calibration curves exhibit a correlation coefficient above the required threshold of *R* ^2^ > 0.99. Additionally, the *F*-value from the ANOVA table exceeds the tabulated value, and the *P*-value is below 0.05 (Table 1). The Anderson-Darling test was applied to assess normality, the Levene test to verify homoscedasticity, and the Breusch-Godfrey test to evaluate residual independence (Table 2). In addition, the residuals were analyzed according to Figure 6.

**Figure 6:**
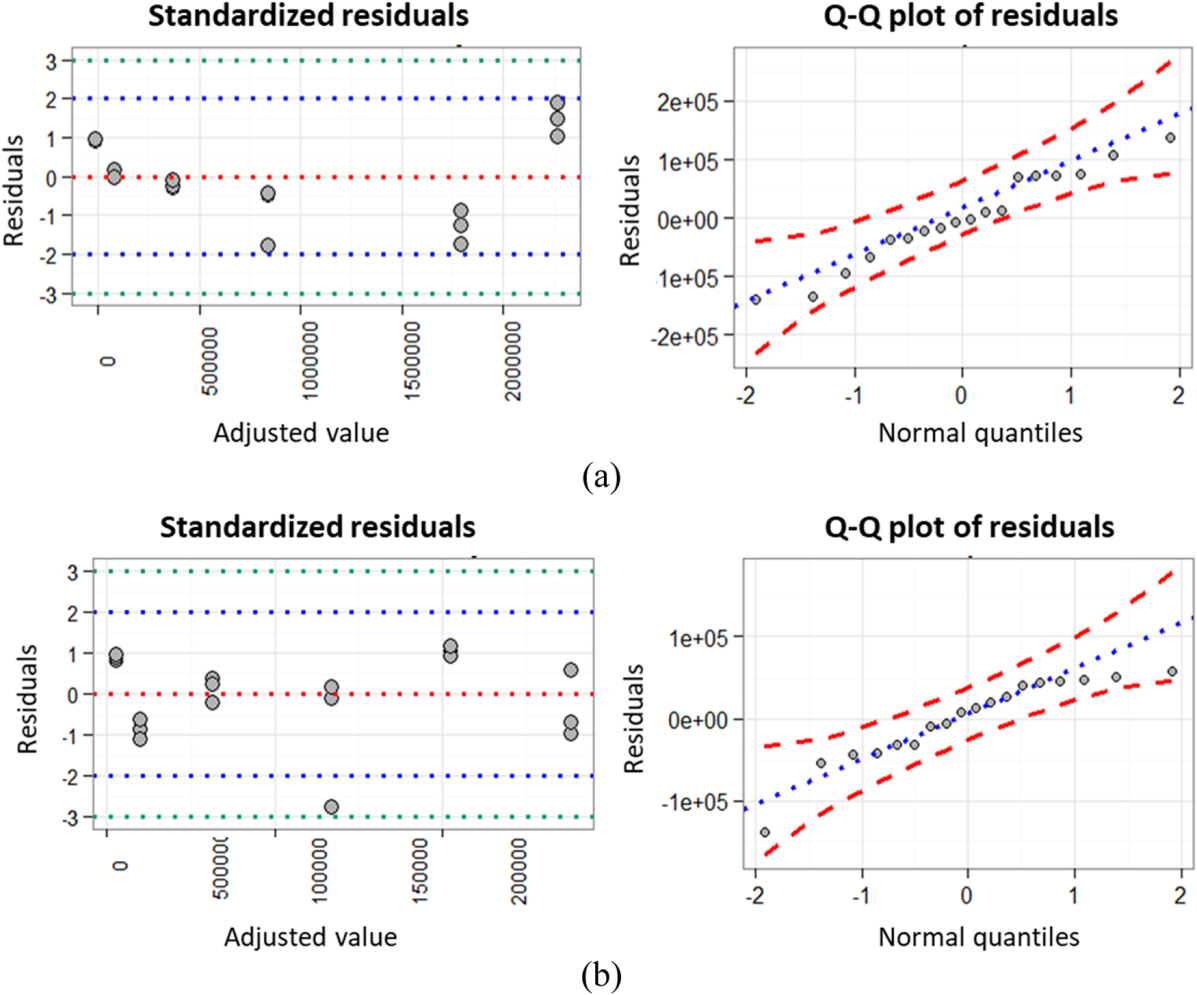
Graphical analysis of linear model residuals for (a) psilocybin and (b) psilocin.

**Table 1:** ANOVA of Calibration Curves of Working Standards of Psilocybin and Psilocin

substance	curve equation/*R* ^2^	ANOVA	DOF^c^	sum of squares	mean square	*F*-value/*P*-value
PSCB	*y* = 18959*x* – 106505^a^	C^b^	1	13,303,299,665,060.1	13,303,299,665,060.1	1993.8
	*R*^2^ = 0.9929	residuals	16	106,754,229,643.4	6,672,139,352.7	0
PSC	*y* = 28507*x* – 86871^a^	C^b^	1	17,481,898,894,698.4	17,481,898,894,698.4	6550.0
	*R*^2^ = 0.9993	residuals	16	42,703,541,952.0	2,668,971,372.0	0

a *y*—Area and *x*—concentration (mg/L).

b Concentrations (mg/L).

c Degrees of freedom.

**Table 2:** Results of Linear Regression Assumption Tests Applied to the Residuals of Psilocybin and Psilocin Models

substance	normality test (Anderson-Darling)	homoscedasticity test (Levene)	independence test (Breusch-Godfrey)
psilocybin	0.629	0.591	0.051
psilocin	0.127	0.520	0.066

The results showed that the residuals from the regression models for both psilocybin and psilocin follow a normal distribution, with *P*-values of 0.629 and 0.127, respectively. Homoscedasticity was confirmed by Levene’s test, which yielded *P*-values of 0.591 for psilocybin and 0.520 for psilocin, indicating no evidence of unequal variances. Regarding independence, the Breusch-Godfrey test returned *P*-values of 0.051 for psilocybin and 0.066 for psilocin. Although the value for psilocybin approaches the 0.05 threshold, it still falls within the acceptable range, suggesting no statistically significant autocorrelation in the residuals.

Thus, it is evident that the constructed calibration curves demonstrate good linearity (Figure 5), effectively correlating chromatographic peak area data with analyte concentration in the matrix across the specified working ranges. The absence of outliers in the data set was confirmed by evaluating the residual plot, which indicates that all values fall within the specified range.

Limits of detection (LOD) and quantification (LOQ) were determined based on the standard deviation of the linear coefficient from calibration curves, following eqs 1 and ([Disp-formula eq2]). The calculated LOD and LOQ values for psilocybin are 1.58 mg/L and 4.78 mg/L, respectively. For psilocin, the LOD and LOQ values are 1.70 mg/L and 5.17 mg/L, respectively.

The LOD and LOQ values obtained for psilocybin and psilocin using HPLC-DAD are consistent with those expected for methods employing diode array detectors, as reported by Perez^20^ which obtained LOD and LOQ values of 1.19 mg/L and 3.6 mg/L, respectively, considering the nature of the analytes and their typical concentrations in *Psilocybe cubensis*.

Although DAD exhibits lower sensitivity compared to mass spectrometry (MS), it remains effective for routine quantification when the method is properly optimized and validated. The choice of DAD reflects a balance between adequate sensitivity, robustness, accessibility, and cost-effectiveness, resulting in a reliable method for the intended purpose. The sensitivity achieved was compatible with the concentration ranges under study and aligned with the analytical objectives. Therefore, although the LOD/LOQ values are higher than those reported for LC-MS^21^ (obtaining LOD and LOQ values of 1.5 and 5.0 mg/L for psilocybin and 0.15 and 0.50 mg/L for psilocin) the method performance was satisfactory for the study requirements and in accordance with the current regulatory standard, ANVISA RDC 166/2017, which guided the development of the method.

### Recovery, Accuracy and Precision

Recovery, precision and accuracy were assessed at three concentration levels (10, 50, and 100 mg/L), with triplicate measurements performed for each level, as detailed in Table 3.

**Table 3:** Recovery, Accuracy and Precision of the HPLC-PDA Method Developed (*n* = 3)

substance	concentration level (mg/L)	mean recovery (%)	repeatability (SD %)	intermediate precision (SD %)	accuracy (%)
PSCB	10	100.7 ± 0.30	3.90	12.75	122.0 ± 3.90
	50	101.8 ± 0.01	1.24	13.57	105.2 ± 1.24
	100	89.1 ± 0.03	0.43	11.86	80.1 ± 0.43
PSC	10	77.5 ± 2.15	2.05	3.34	116.0 ± 2.05
	50	88.5 ± 0.74	1.60	2.43	98.0 ± 1.60
	100	100.8 ± 0.59	0.10	0.49	98.0 ± 0.10

Psilocybin recovery ranged from 89.1 to 101.8% across the three concentration levels tested, indicating acceptable precision according to validation criteria that often consider 80–120% as the acceptable range for recovery, particularly in complex matrices.^22,23^ At concentrations of 10 and 50 mg/L, recoveries were slightly above 100%, suggesting a negligible positive matrix effect or variability in detector response. At concentrations of 100 mg/L, recovery dropped to 89.1%, still within acceptable limits. This small reduction may be attributed to minor saturation effects in the extraction phase or losses during the drying/reconstitution steps, which are more pronounced at higher analyte concentrations. Standard deviation (SD) values remained low across all concentrations, with CVs below 3.0%, indicating excellent method reproducibility and consistency.

The recovery profile of psilocin shows a progressive improvement with increasing concentration. The low standard deviation observed (SD < 2.2 across all levels) demonstrates, however, that although recovery was slightly lower at 10 mg/L, the method was accurate and consistent. At 100 mg/L, the recovery reached 100.8%, indicating excellent precision with low variability. However, at 10 mg/L, the recovery was 77.5%, slightly below the commonly accepted limit of 80%. This result may reflect a greater susceptibility of psilocin to degradation or adsorption at lower concentrations. Psilocin is known to be less stable than psilocybin due to its hydroxyl group at the 4-position of the indole ring, which is easily deprotonated and increases its reactivity in aqueous and oxidative conditions.

The repeatability, or intraday precision, demonstrated a relative standard deviation (SD) ranging from 0.43 to 3.90% for psilocybin and 0.10 to 2.05% for psilocin, indicating low variability. It is important to note that due to the independent nature and lack of correlation between the data for psilocybin and psilocin, differences in variability intervals for repeatability, accuracy, and intermediate precision are natural.

For intermediate precision, also known as interday precision, the SD values ranged from 11.86 to 13.57% for psilocybin and from 0.49 to 3.34% for psilocin. The difference between the observed values for these molecules can be attributed to the fact that the data for each substance were independently used in developing their respective mathematical models. Regarding the method’s accuracy, the recovery intervals for the analytes ranged from 80 to 120% for psilocybin, whereas for psilocin, recovery intervals ranged of 98 to 116%. According to ANVISA guidelines, the acceptable accuracy range is between 80% and 120%, indicating that the result at the 100 μg/mL level falls within acceptable limits of variation.

### Matrix Effect

To assess the impact of other compounds present in the analyte matrix (extract) on the linearity of the developed calibration curve, it was necessary to determine the presence or absence of this effect. This was achieved by comparing the calibration curve (Figure 5) constructed solely with analytical standards against the curve constructed by fortifying the matrix with these standards. Analysis of the peak areas of the substances with and without the matrix revealed noncoincident lines. Moreover, both substances showed correlation coefficients (R^2^) above 0.99 when analyzed with the matrix.

To further investigate potential matrix effects, a new approach was employed using independent calibration curves prepared in triplicate with and without the matrix. For each replicate, the slope and intercept were obtained and compared using a paired two-sample *t* test and parallelism by ANOVA F-test (Table 4). The calibration curves obtained with and without the matrix are depicted in Figure 7.

**Figure 7:**
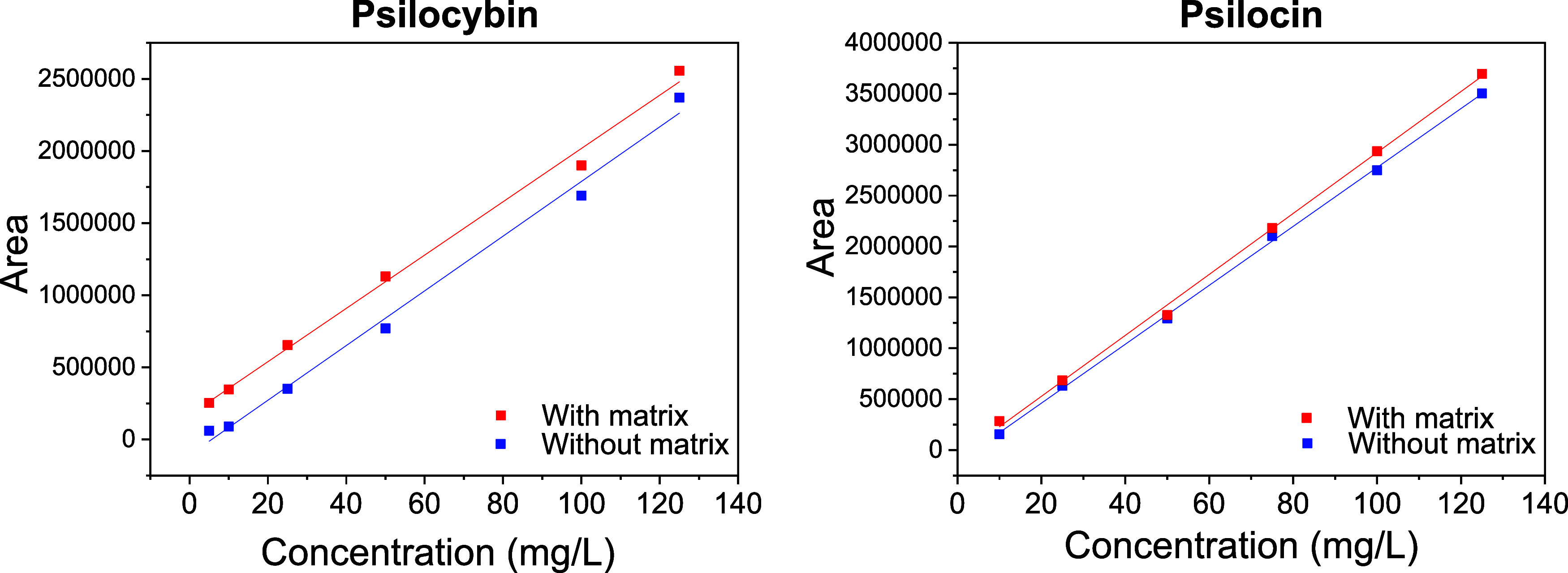
Calibration curves with and without matrix for psilocybin and psilocin.

**Table 4:** *P*-values for Matrix Effect Significance, Equality of Intercept, and Linearity of the Lines

substance	evaluation	statistic	*P*-value
PSCB	intercept comparison (*t* test)	0.926	0.452
	parallelism (ANOVA, test *F*)	0.769	0.387
	slope comparison (*t* test)	2.387	0.139
PSC	intercept comparison (*t* test)	0.490	0.672
	parallelism (ANOVA, test *F*)	1.641	0.209
	slope comparison (*t* test)	0.987	0.427

For psilocybin, the intercept comparison yielded a *t*-value of 0.926 with a *P*-value of 0.452, and the slope comparison produced a *t*-value of 2.387 with a *P*-value of 0.139. In both cases, the *P*-values exceeded the significance threshold (α = 0.05), indicating no statistically significant differences between the calibration curves constructed with and without matrix. The parallelism test via ANOVA (*F* = 0.769; *p* = 0.387) further confirmed the absence of significant deviation between slopes, suggesting no matrix effect on the calibration curve for this analyte. For psilocin (PSC), the intercept comparison (*t* = 0.490; *p* = 0.672) and slope comparison (*t* = 0.987; *p* = 0.427) likewise revealed no statistically significant differences. The parallelism test (*F* = 1.641; *p* = 0.209) supported these findings, indicating that the calibration curves are statistically equivalent regardless of matrix presence.

Collectively, these results confirm that the developed method is not significantly influenced by the matrix and is therefore suitable for accurate quantification of psilocybin and psilocin in complex sample matrices, including biological or phytopharmaceutical preparations.

### Working Range

The working range (WR) was calculated using eq 4, where the theoretical concentration (*C*_theo_) corresponds to the proposed concentration for each injection (5, 10, 25, 50, 75, 100, and 125 mg/L). The extract concentration (*C*_ext_) was obtained experimentally by injecting a solution containing 1 mg/mL of the extract and the total concentration was determined from the area obtained in the injections performed with the enrichment of the matrix, through eqs 5 and 6.

5 $$C=\frac{A_{PSCB}+106,505}{18,959}$$

6 $$C=\frac{A_{\mathrm{PSC}}+86,871}{28,507}$$

where *C* represents the desired concentration the extract for the area of psilocybin (*A*_PSCB_) or psilocin (*A*_PSC_) obtained from the chromatograms, obtained for injections of the extract and extract enriched with the standards (total). The calculated working ranges for psilocybin and psilocin are presented in Table 5.

**Table 5:** Working Range Calculated for Psilocybin and Psilocin

concentration (mg/L)	5	10	25	50	75	100	125
W*R*_PSCB_	95.04	100.74	103.68	101.82		89.09	99.17
W*R*_PSC_	75.83	77.50	87.25	88.55	99.03	101.63	

According to the adopted standard, the working range should remain between 80 to 120%. It was noted that all tested concentration points for psilocybin are within the values stipulated by the standard. However, for psilocin, the lower concentration points are more susceptible to measurement precision, as they approach the lower limits of detection, occasionally appearing outside the specified range, though with minimal variation. Consequently, the method for psilocin maintains a suitable working range starting from 10 mg/L.

### Robustness

To assess of the method robustness, three variables were examined: pH of the mobile phase, column oven temperature, and batches of solvent from different manufacturers (Table 6). Furthermore, accuracy assay concentration levels were evaluated, as stipulated by RDC No. 166/2017 of ANVISA (Brazil). The outcomes of all variations were subsequently compared with those of the unmodified method.

**Table 6:** Conditions for Assessing Method Robustness

concentration (mg/L)	temperature (°C)	pH (acidification of FM)	supplier ACN
10, 50 e 100	30	0.3% formic acid	supplier A
10, 50 e 100	40	0.3% formic acid	supplier A
10, 50 e 100	30	0.2% formic acid	supplier A
10, 50 e 100	30	0.3% formic acid	supplier B

The effects of method condition changes on peak areas at various concentration levels were evaluated. The Half-Normal and Lenth methods were employed to estimate the magnitude of these effects for each variable (Table 7) and establish acceptable ranges of variation in the results (Table 8).

**Table 7:** Estimation of Effect Magnitude

substance		effects	estimate	inferior limit	upper limit	*t*-value	*P*-value
PSCB	intercept		147,668.29				
	P10	154,913.04	77,456.52	–6,930,269.13	7,240,095.21	0.0629	0.9531
	P11	2,595,796.83	1,297,898.41	–4,489,385.34	9,680,979.01	1.0547	0.356
	P12	5,681,622.70	2,840,811.35	–1,403,559.47	12,766,804.87	2.3086	0.0882
	P2	1,508,995.17	754,497.58	–5,576,187.00	8,594,177.35	0.6131	0.5758
	P3	2,535,994.02	1,267,997.01	–4,549,188.15	9,621,176.19	1.0304	0.3659
	P4	–91,212.38	–45,606.19	–7,176,394.56	6,993,969.78	0.0371	0.9724
	P5	986,462.51	493,231.25	–6,098,719.66	8,071,644.68	0.4008	0.7108
	P6	1,865,211.56	932,605.78	–5,219,970.61	8,950,393.73	0.7579	0.4943
	P7	18,306.86	9,153.43	–7,066,875.31	7,103,489.034	0.0074	0.9945
	P8	1,640,727.57	820,363.78	–5,444,454.60	8,725,909.75	0.6667	0.5446
	P9	3,767,288.29	1,883,644.14	–3,317,893.88	10,852,470.46	1.5307	0.2069
PSC	intercept		218,040.18				
	P10	19,148.62	9,574.313	–9,904,806.35	9,943,103.60	0.0056	0.9959
	P11	2,298,106.97	1,149,053.48	–7,625,847.99	12,222,061.94	0.6667	0.5446
	P12	5,388,636.71	2,694,318.35	–4,535,318.26	15,312,591.68	1.5632	0.1994
	P2	2,161,332.54	1,080,666.27	–7,762,622.43	12,085,287.51	0.627	0.5676
	P3	5,006,954.44	2,503,477.22	–4,917,000.53	14930909.41	1.4525	0.2262
	P4	–34,151.71	–17,075.85	–9,958,106.68	9,889,803.26	0.0099	0.9926
	P5	2,173,806.02	1,086,903.01	–7,750,148.95	12,097,760.99	0.6306	0.5654
	P6	5,236,258.18	2,618,129.09	–4,687,696.79	15,160,213.15	1.519	0.2097
	P7	17,927.86	8,963.93	–9,906,027.10	9,941,882.83	0.0052	0.9961
	P8	2,320,647.16	1,160,323.58	–7,603,307.80	12,244,602.13	0.6732	0.5408
	P9	5,508,115.67	2,754,057.83	–4,415,839.29	15,432,070.64	1.5979	0.1917

**Table 8:** Limits Calculated by the Lenth Method

substance	α	PSE	ME	SME	t.crit
PSCB	0.05	2,461,091.36	7,085,182.17	15,177,111.70	2.88
PSC	0.05	3,447,160.46	9,923,954.97	21,258,024.07	2.88

Since the *P*-values of the Student’s *t* test are greater than 0.05, we reject the hypothesis of null effects at the 5% significance level. For this method, the Pseudo Standard Error (PSE) is calculated. From this, a margin of error (ME) is defined. Contrasts that exceed the ME value, in absolute value, are considered significant. A simultaneous margin of error (SME) is also defined, since, if there is a large level of contrasts, it is expected that some estimates of nonsignificant contrasts exceed the ME value. The value of t.crit in the Table (2.88) indicates the cutoff point above which the contrasts can be considered significant at the level of α = 0.05.

Through these analyses, it was observed that none of the concentration points exhibited significant differences compared to the results obtained with the unmodified method (i.e., all points had *p* > 0.05). Furthermore, when evaluating the estimates graph (Figure 8) using the limits calculated in Table 8, no effect values were found to exceed these limits. Therefore, changes in the method’s conditions do not significantly impact the results, indicating the robustness of the method.

**Figure 8:**
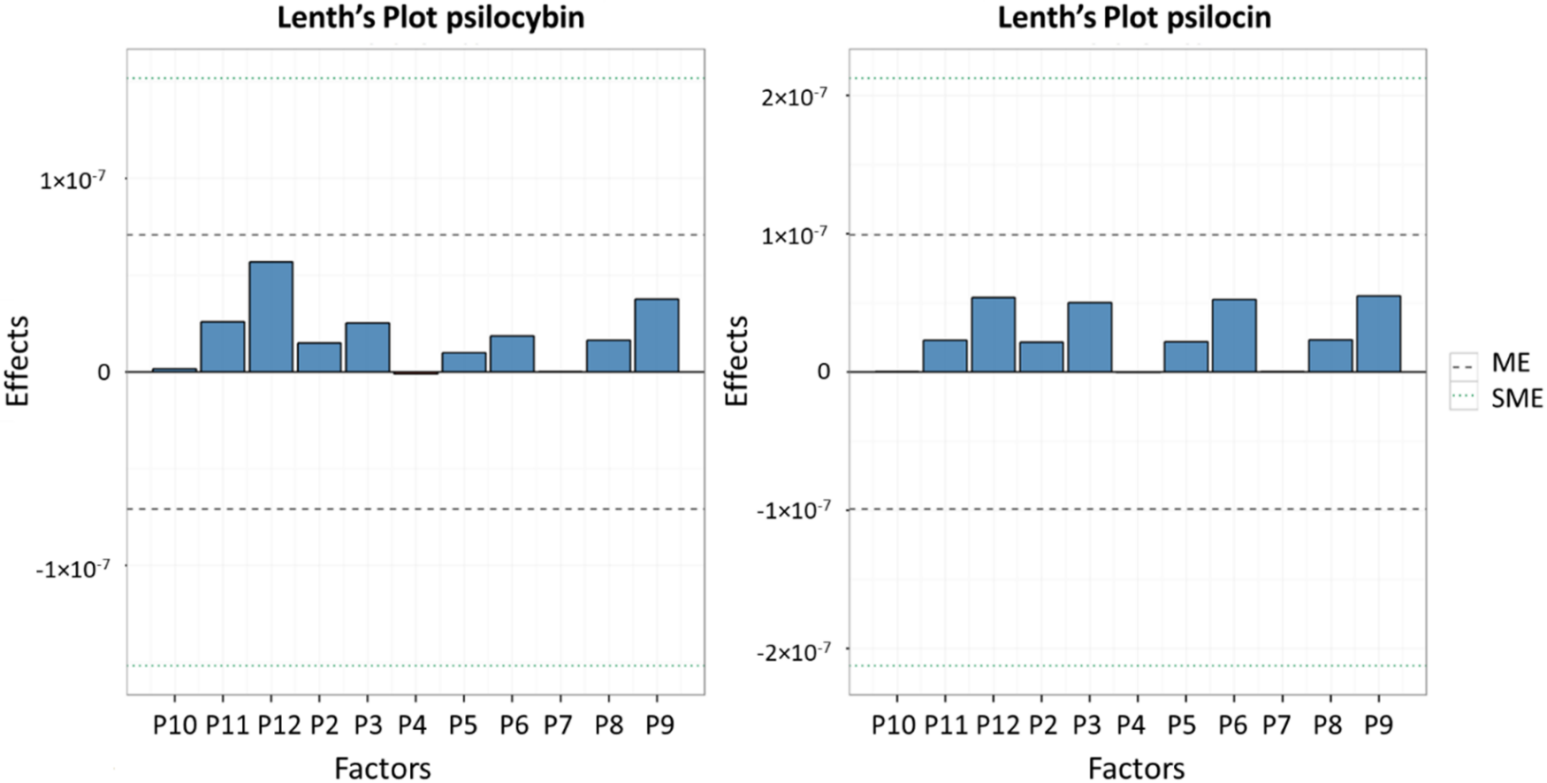
Lenth plot with error margins for psilocybin and psilocin.

The high-performance liquid chromatography with diode array detector (HPLC-DAD) method developed for the quantification of psilocybin and psilocin in psychedelic mushroom extracts demonstrated high specificity, satisfactory sensitivity, and optimization of analysis time. When compared to more advanced techniques, such as mass spectrometry (LC-MS) and fluorescence detection, HPLC-DAD has advantages and limitations that should be considered. Its main advantage lies in the accessibility of the equipment and operational simplicity, in contrast to mass spectrometry, which, although more sensitive and specific, requires sophisticated infrastructure and greater financial investment.^24^

In addition, the reduction in chromatographic run time and solvent consumption in the developed protocol optimizes the separation of analytes, making it an economically viable option. However, its detection capacity may be lower than that obtained by LC-MS, impacting the analysis of samples with low concentrations of the target compounds.

### Application of the Method

The developed chromatographic method aims to accurately identify and quantify psilocybin and psilocin in extracts from *Psilocybe cubensis* mushrooms. Based on analytical validation, the method was effective and efficient to identify and quantify psilocybin and psilocin, meeting all the necessary criteria. Therefore, extractions were conducted using mushrooms of the *Psilocybe cubensis* species and subsequently analyzed using the developed methodology.

The extract was prepared and dissolved in three solutions with the same composition as the initial step of the chromatographic method, reaching a concentration of up to 2.5 mg/L. Following analysis, chromatograms were obtained (Figure 9), and the peak areas used for quantifying the active compounds were extracted, in order, to determine the concentrations of psilocybin and psilocin present (Table 9).

**Figure 9:**
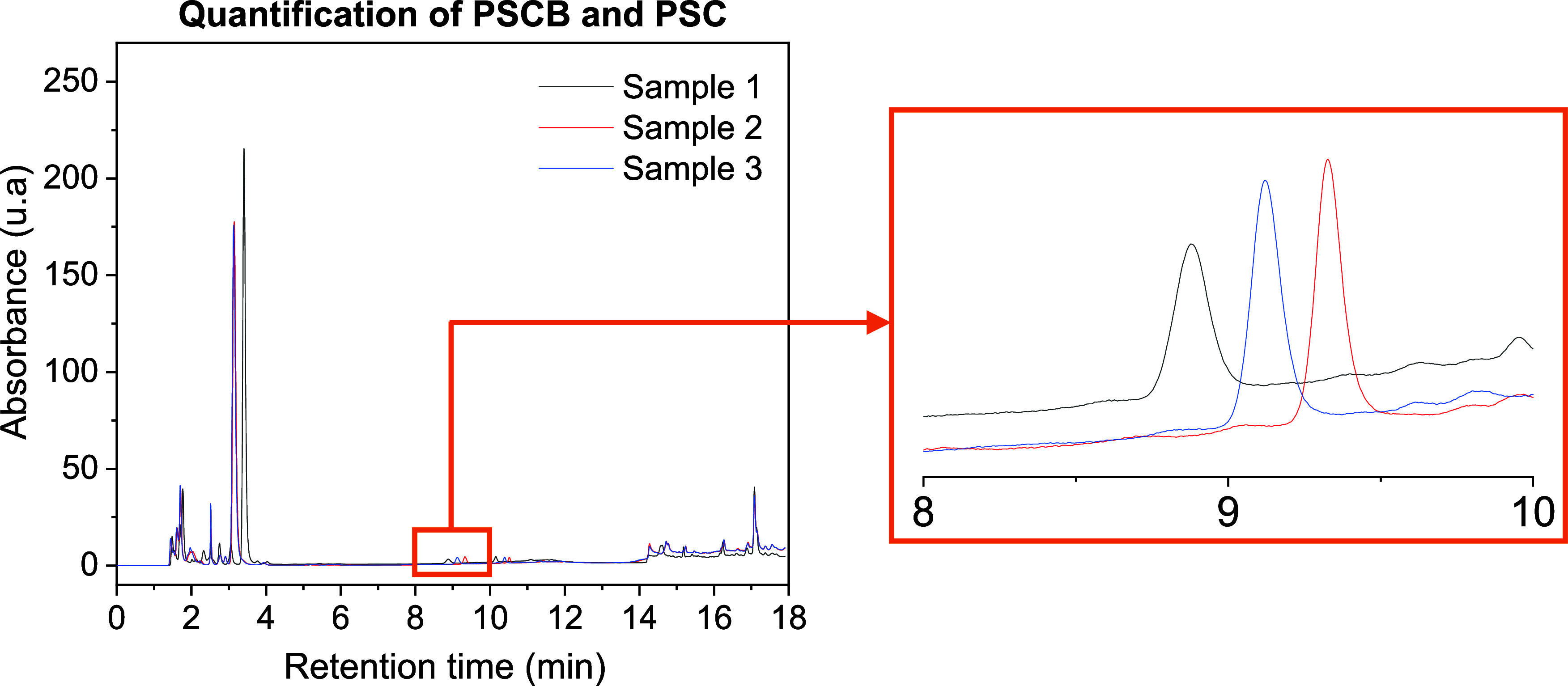
Chromatograms obtained from injections performed at a wavelength of 266 nm for three samples prepared under identical extraction and cleanup conditions, aimed at quantifying psilocybin (PSCB) and psilocin (PSC).

**Table 9:** Concentration (in Percentage) of Psilocybin and Psilocin in the Extracts

substance	sample 1 (%)	sample 2 (%)	sample 3 (%)	average (%)
psilocybin	2.58	2.56	2.56	2.57 ± 0.01
psilocin	0.15	0.16	0.16	0.16 ± 0.01

The average concentration of psilocybin (PSCB) in the extracts was approximately 2.57%, while psilocin (PSC) was present at about 0.16%. These findings are corroborated by Galdino et al.,^25^ who reviewed extraction yields of psilocybin and psilocin across several studies. The review confirms that psilocybin typically occurs at higher concentrations than psilocin, a pattern attributed to its greater chemical stability. Psilocin is more susceptible to degradation under environmental stressors such as oxygen, heat, and light.^17,26^

In this study, the observed psilocybin content (2.57%) falls within the upper range of values reported in the literature. Notably, previous studies have also documented psilocybin yields exceeding 2.5% in dry mushroom samples,^27,28^ demonstrating that such high concentrations, while less frequent, are consistent with well-documented occurrences. These elevated values can be attributed to a combination of factors, including optimized cultivation conditions, the genetic potential of specific *Psilocybe* strains, and efficient extraction methodologies. Environmental variables such as the pH and composition of the cultivation substrate, mushroom maturity at harvest, temperature, humidity, and light exposure directly influence the biosynthesis of indole alkaloids. For instance, mushrooms harvested at full maturity generally accumulate higher levels of psilocybin. Furthermore, precise control of temperature and humidity enhances alkaloid production, while light plays a critical role in fruiting body development.^28,29^ Differences in extraction techniques and analytical procedures across studies may also contribute to the observed variability. Taken together, these factors support the plausibility and reproducibility of the high psilocybin yield obtained in the present study, reinforcing the reliability of our results and the robustness of our experimental approach.

## Conclusions

In this study, a rapid and reliable extraction protocol was employed for the purpose of extracting psilocybin from magic mushrooms. In addition, a robust and validated HPLC-DAD methodology was developed to detect and quantify psilocybin and psilocin in extracts from psychedelic mushrooms intended for medicinal use. Following the guidelines of RDC No. 166/2017 from the Brazilian National Health Surveillance Agency (ANVISA), we successfully validated an HPLC method employing a PerkinElmer C18 column under specific gradient conditions using ultrapure Milli-Q water and acetonitrile acidified with formic acid. The method exhibited limits of detection and quantification values of 1.58 and 4.78 mg/L for psilocybin, and 1.70 and 5.17 mg/L for psilocin, respectively. While it is acknowledged that techniques such as LC-MS may offer lower detection limits, the sensitivity achieved with the DAD detector was shown to be adequate for the concentration ranges relevant for the purpose of this study. Accuracy assessments demonstrated recovery intervals of 80–120% for psilocybin and 98–116% for psilocin, critical for their pharmaceutical application. Precisely quantifying psilocybin (2.57%) and psilocin (0.16%) is essential for ensuring their efficacy and safety in therapeutic settings. It should be noted, as a general consideration for chromatographic methods in complex matrices, that potential matrix effects may influence the results, although the validation data from this study met the acceptance criteria. This robust HPLC methodology represents a significant advance in the accurate measurement of these compounds, facilitating their future use to precisely quantify the substances for the development of reliable pharmaceutical formulations for clinical research and therapeutic practice. Furthermore, future studies can expand the analysis of psilocybin analogues, standardize their potency for clinical applications, and investigate factors that influence their stability and variability. In addition, research on cultivation conditions and genetic cloning can reduce inconsistencies in the composition of mushrooms.
